# Vitamin D Status Across Age Groups in Turkey: Results of 108,742 Samples from a Single Laboratory

**DOI:** 10.4274/jcrpe.galenos.2019.2019.0097

**Published:** 2020-09-02

**Authors:** Gül Yeşiltepe-Mutlu, Ekin Deniz Aksu, Abdullah Bereket, Şükrü Hatun

**Affiliations:** 1 Koç University Faculty of Medicine, Department of Pediatric Endocrinology, İstanbul, Turkey; 2 Marmara University Faculty of Medicine, Department of Pediatric Endocrinology, İstanbul, Turkey

**Keywords:** Vitamin D, deficiency, National Prophylaxis Program, 1 year of age

## Abstract

**Objective:**

The aim was to determine vitamin D status in the general population in Turkey between 2011 and 2016, and to evaluate the effectiveness of the national vitamin D supplementation programme.

**Methods:**

Serum 25-hydroxyvitamin D (25-OHD) measurement data were retrieved from an internationally accredited laboratory, operating nationwide. A total of 108,742 measurements of 25-OHD were analyzed using the cut-off values of 0-11 ng/mL, 12-19 ng/mL, 20-49 ng/mL, 50-70 ng/mL and >70 ng/mL for vitamin D deficiency, insufficiency, sufficiency, possibly harmful and excess respectively.

**Results:**

The mean±standard deviation 25-OHD level was 21.6±13.3 ng/mL. Mean 25-OHD concentrations by age groups were: 37.3 ng/mL, 30.1 ng/mL and 23.7 ng/mL for <1, 1-10 and 11-18 year old groups, respectively. Mean 25-OHD levels of children <1 year and 1-3 years of age were significantly higher than those found in other age groups. The prevalence of vitamin D deficiency (<12 ng/mL) was lowest in children at 1-3 years of age (5%). In subjects older than 18 years of age, mean 25-OHD levels were 18.2 ng/mL, 20.1 ng/mL, 21.9 ng/mL and 21.1 ng/mL for age groups 19-30, 31-50, 51-70 and >70 years, respectively.

**Conclusion:**

Successful implementation of the national vitamin D supplementation programme, appears to have nearly eliminated vitamin D deficiency for children under 1-years of age. However, the positive impact of the vitamin D supplementation diminishes as children get older suggesting that supplementation may be required in the older children and adults. In addition, improved awareness of the benefits and risks of excess vitamin D should prevent unnecessary and excessive use of vitamin D supplements.

**What is already known on this topic?**

The extra-skeletal effects of vitamin D have caused an increase in interest in vitamin D. This interest has led to an increase in requests for serum 25-hydroxyvitamin D (25-OHD) levels. Previous reports have suggested a high prevalence of vitamin D deficiency in Turkey. Furthermore, high dose vitamin D treatment has been prescribed, even in cases with normal 25-OHD levels, in order to obtain some of the extra-skeletal effects of vitamin D.

**What this study adds?**

This study with a very large sample size aimed to assess vitamin D status in Turkey. Data from this study do not support the idea of a vitamin D deficiency pandemic in Turkey. Successful implementation of the vitamin D supplementation program appears to have overcome vitamin D deficiency for those under 1-year of age in Turkey.

## Introduction

In the past 10 years, there has been growing interest in vitamin D deficiency, especially concerning its extra-skeletal effects. Serum 25-hydroxyvitamin D (25-OHD) measurements have become a part of routine health evaluations in both pediatric and adult populations, and more importantly, claims about a “vitamin D deficiency pandemic” have generated considerable debate associated with diverse definitions of deficiency (1). According to a recently published study evaluating data from 711,718 children from Britain, the incidence of vitamin D deficiency increased from 3.14/100,000 in 2000 to 261/100,000 in 2014, amounting to a 15-fold increase after adjusting for population increases (2).

In Turkey, data are lacking on annual changes in the incidence of vitamin D deficiency. According to the “Intercontinental Marketing Services Health” database, in 2012, a total of 2,280,626 ampoules of vitamin D (containing 300,000 international units per ampoule) were sold in Turkey, with about 4 times increase to 8,376,319 ampoules in the first eight months of 2016. This is despite a nationwide vitamin-D-prophylaxis programme, including free provision of vitamin D drops (400 international units daily) to infants, which has successfully operated since 2005 (3).

This population-based study aimed to determine the vitamin D status in Turkey between 2011 and 2016, evaluate the effectiveness of the vitamin D supplementation programme which started in 2005, analyze the relationships among simultaneous serum measurements of the 25-OHD, parathyroid hormone (PTH) and alkaline phosphatase (ALP), and determine the prevalence of vitamin D deficiency by age, gender, year, region and season.

## Methods

Data on serum 25-OHD measurements between January 2011 and December 2016 were retrieved from an internationally accredited private laboratory operating nationwide in Turkey.

No ethical approval was sought as this study was a retrospective study of previously collected data. In addition no consent form was issued although approval from the owners of the data was obtained.

Serum 25-OHD concentrations were measured with the liquid chromatography-tandem mass spectrometry (LC-MS/MS) method, Quattro Premier QE (Waters, MA, USA), as the sum of D2 and D3 levels. After adding internal standard (Deuterated stable isotope) to the calibrators (Chromsystems 3PLUS1 multilevel Serum calibrator set 25-OH-VITAMIN D3 and D2), controls (Chromsystems Level 1/2 25-OH-VITAMIN D3 and D2) and samples, extraction with hexane is performed. After extraction, the extracts dissolved in 70% methanol are injected into the LCMS/MS device. Acquity BEH 2.1x50 mm C8 1.7 µm column was used in the analysis. The measurement range of LC-MS/MS method for 25-OHD concentration was 2.5-100 ng/mL. Serum PTH concentrations were measured by an electrochemiluminescence immunoassay (ECLIA) and serum ALP concentrations were measured by a colorimetric assay using the Roche Cobas e601 and Cobas c501 modules (Roche Diagnostics, Mannheim, Germany), respectively, in accordance with the International Federation of Clinical Chemistry (IFCC) standardization.

A total of 108,742 random measurements of 25-OHD were analyzed according to age, gender, region, season and year, using the cut-off values of 0-11 ng/mL, 12-19 ng/mL, 20-49 ng/mL, 50-70 ng/mL and >70 ng/mL from the 2001-2006 data of the National Health and Nutrition Examination Survey (NHANES) (4). According to the Global Consensus Recommendations, a serum 25-OHD concentration of less than 12 ng/mL was considered to be diagnostic for vitamin D deficiency, while concentrations between 12-20 ng/mL were considered vitamin D insufficiency, and concentrations above 20 ng/mL but less than 50 ng/mL were considered normal (5). In addition, 25-OHD concentrations greater than 50 ng/mL were defined as ‘possibly dangerous’ according to the recommendations of the Centers for Disease Control and Prevention (CDC) (4). Of the total sample of 108,742, 17% (n=18,613) were samples obtained from patients younger than 18 years of age. Repeat measurements obtained from the same individuals at different times were excluded from the study.

The data were compared with those of the NHANES 2001-2006 reports. Moreover, correlations between 25-OHD and ALP or PTH were assessed when simultaneous ALP (n=10,139) or PTH (n=4,916) measurements were available.

### Statistical Analysis

The statistical analysis was performed using R program. Outliers were discarded using Tukey’s method with boundaries 2.5 times the interquartile range (IQR), which amounted to 25-OHD values between 0.25 ng/mL and 74.5 ng/mL. A total of 2040 observations were discarded, all of which were between 74.5 ng/mL and 1590 ng/mL. The Wilcoxon test was used for between-group comparisons of 25-OHD. The Kruskal-Wallis test followed by the Conover post-hoc test was used for multiple comparisons. The Spearman’s correlation coefficient was used for bivariate correlations, and multiple comparisons were corrected using Bonferonni’s method.

## Results

**Figure 1:**
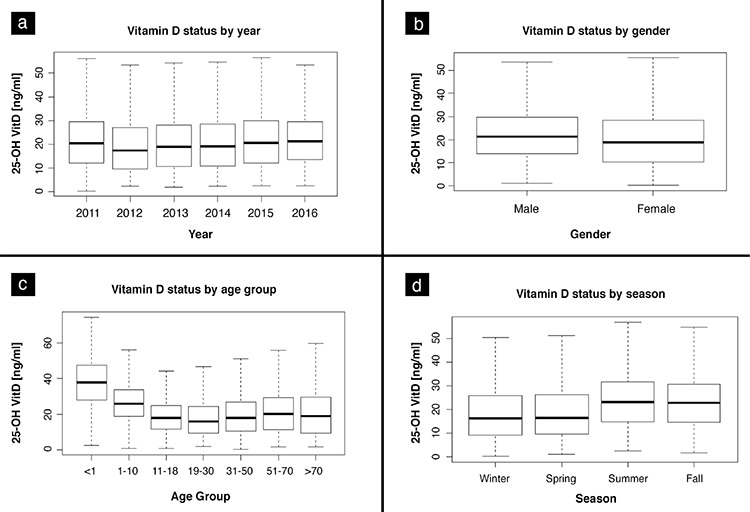
Distribution of serum 25-OHD levels according to years (a), gender (b), age groups (c), and seasons (d) (ng/mL) 25-OHD: 25-hydroxyvitamin D, VitD: vitamin D

The number of 25-OHD measurements was similar across years, with a female predominance (72.9%). Overall, the mean±standard deviation 25-OHD concentration was 21.6±13.3 ng/mL, being lower in women (21±13.5 ng/mL) than in men (23.2±12.5 ng/mL) (p<0.001). The mean 25-OHD concentrations according to age groups were as follows: 37.2 ng/mL for <1 year of age, 27.1 ng/mL for 1-10 years of age, 19.2 ng/mL for 11-18 years of age, 18.25 ng/mL for 19-30 years of age, 20.1 ng/mL for 31-50 years of age, 21.9 ng/mL for 51-70 years of age, and 21.1 ng/mL for >70 years of age. The mean 25-OHD levels among children <1 year of age and 1-10 years of age were significantly higher compared with those found for the other age groups (p<0.001). According to seasonal distribution, 25-OHD concentrations were higher in summer (23.8 ng/mL) and fall (24.5 ng/mL) than those measured in winter (19.1 ng/mL) and spring (19.6 ng/mL) (p<0.001). Serum 25-OHD levels according to years, seasons, geographic regions, age groups, and gender are shown in Table 1 and Figure 1.

**Figure 2:**
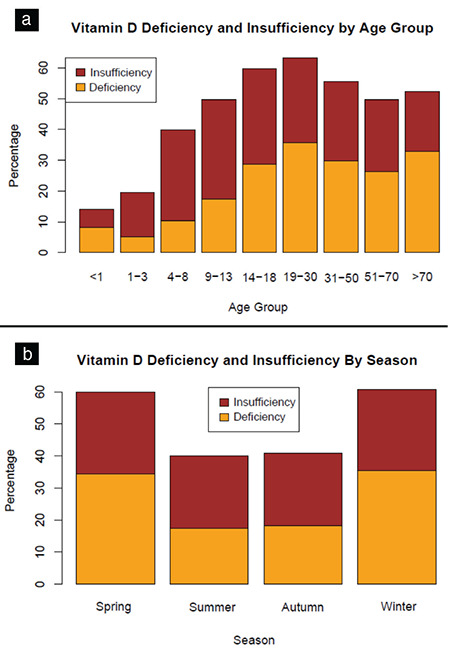
The prevalence rates of vitamin D deficiency and insufficiency according to age groups (a) and seasonal distribution (b) (%)

The prevalence of vitamin D deficiency (<12 ng/mL) was lowest in children under 1 year of age (7%) and 1-10 years of age (8%), while the highest rates of prevalence were noted in women (30%), in those 19-30 years of age (36%), in the Black Sea region (42%), and in winter (36%). The prevalence rates of vitamin D deficiency and insufficiency according to age groups and seasonal distribution are shown in Figure 2.

**Figure 3:**
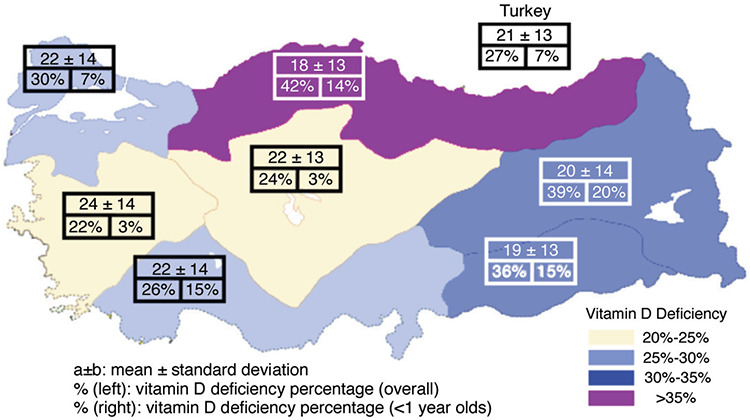
Regional distribution of the mean 25-hydroxyvitamin D levels (ng/mL) and the frequencies of vitamin D deficiency (overall and for <1 year of age) (%)

**Figure 4:**
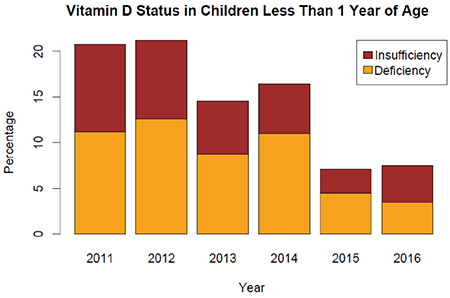
The prevalence rates of vitamin D deficiency and insufficiency for the youngest age group (<1 year) according to years

For the youngest age group (<1 year), the prevalence of vitamin D deficiency was highest in the Mediterranean (15%), Black Sea (14%) and Southeast (15%) regions (Figure 3). For the youngest age groups (<1 year), the rates of deficiency and insufficiency showed decreases over the years and were around 3-4% in 2015 and 2016 (Figure 4). When 25-OHD levels were classified according to the Endocrine Society’s guideline (6) recommending cut-off levels of <20, 20-30 and 30-100 ng/mL for deficiency, insufficiency and sufficiency, respectively, the rate of sufficiency was still highest in the youngest age group (Table 2).

**Figure 5:**
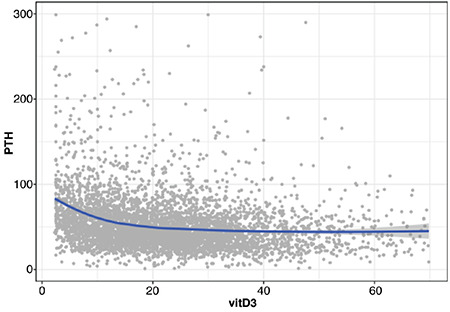
Relation between serum 25-hydroxyvitamin D concentrations and mean (±SE) serum concentrations of parathyroid hormone SE: standard error, vitD: vitamin D, PTH: parathyroid hormone

ALP was simultaneously measured in 9.3%, and PTH was measured in 4.5% of the subjects undergoing serum 25-OHD measurements making correlation analyses possible. In correlation analysis, 25-OHD showed an inverse correlation with PTH in those under 1 year of age (r=-0.28, p<0.001), and those between 11 and 18 years of age (r=-0.38, p<0.001). It was shown that elevated PTH consistently dropped to a plateau when serum 25-OHD was at 20 ng/mL or higher overall (Figure 5). Correlation of serum 25-OHD concentrations with ALP was significant only in the adult population (>18 years) (r=-0.095538, p=0.015). The mean PTH level was significantly elevated in the group with 25-OHD concentrations of 0-20 ng/mL (p<0.0001). However, the mean ALP level was similar across the different vitamin D status groups (p>0.05). Serum concentrations of ALP and PTH according to vitamin D status are shown in Table 3.

## Discussion

Notwithstanding the fact that the present data are from subjects whose clinical features are unknown, 25-OHD measurements indicate the expected differences between genders and seasons in this cohort. More importantly, these results show the beneficial effects of the vitamin D supplementation programme in the 0-1 years-old age group in Turkey, serum 25-OHD levels being highest in the youngest age group, making these data reliable for evaluation. Furthermore, a high data count of 108,742 strengthens the statistical power of the study.

These findings demonstrate a relatively stable number of vitamin D test requests from physicians between 2011 to 2016. It is notable that physicians usually ordered 25-OHD tests without simultaneous ALP or PTH measurements. The growing interest in measuring and use of vitamin D supplementation mainly stems from concerns for the extra-skeletal effects of vitamin D and controversies on the thresholds of serum 25-OHD (7,8). Based on our observations, we think that, due to this interest, physicians often order a 25-OHD test as part of the routine health assessment, especially in women, although it is unlikely that a vitamin D deficiency-related clinical problem was the source of the request. This is the case not only for adults but also for children. In order to prevent unnecessary vitamin D testing in primary care, the Turkish Ministry of Health vitamin D Scientific Board published a guideline about the indications of vitamin D testing (9). In the UK, a 17-fold increase in vitamin D testing and prescription costs, from £1,647 in 2008 to £28,913 in 2014 per 100,000 patient-years has been reported (10), perhaps increased testing leads a dramatic increase in the diagnosis of vitamin D deficiency as well. The overvaluing of 25-OHD measurements without accompanying ALP or PTH measurements (11) and diagnosing ‘vitamin D deficiency’ based on diverse thresholds have resulted in an increased use of high-dose vitamin D (12). However, the Institute of Medicine has emphasized that studies concerning vitamin D and extra-skeletal problems, such as cancer, cardiovascular diseases, diabetes and autoimmune diseases, do not provide consistent evidence and that there is no need to determine varying vitamin D thresholds for these diseases, and thus to prescribe more vitamin D (7,8,13).

In the present study, the mean 25-OHD concentrations found for diverse age groups correspond to those reported in the NHANES 2001-2006 data (Table 4) (4). This similarity may be related to the fact that the samples were obtained from a private laboratory, serving people with relatively high socio-economic status. Nevertheless, when the same thresholds are used, a notably higher prevalence of vitamin D deficiency in Turkey becomes apparent (27% vs. 8%), mostly owing to adults with Vitamin D deficiency, which might be related to less exposure to sunshine due to clothing styles and less intake of dairy products in Turkey. Moreover, vitamin D insufficiency still appeared to be a health issue, particularly among the adults. The prevalence rates of vitamin D deficiency and insufficiency were found to be 27% and 24%, respectively, with increasing rates in the less economically developed regions such as Eastern Anatolia (39%) and Southeastern Anatolia (36%), and in the Black Sea region (42%) which has a relatively low number of sunny days, which supports that hypothesis. However, a very recent retrospective study (14) from the western part of Turkey showed the prevalence of vitamin D insufficiency and deficiency among children to be 21.3% and 44.8%, respectively. Although these proportions are higher than those in our study, the highest proportion having normal vitamin D status was found in the 0-1 years of age group, in keeping with our results.

Another interesting finding was that a 25-OHD level of greater than 50 ng/mL, which the CDC defines as “possibly harmful”, was found in 4% of subjects compared with 1% in the United States. Although we do not have definitive data to account for this discrepancy, this might be related to the recently increased use of vitamin D ampoules in Turkey which contain 300,000 IU of vitamin D per ampoule, instead of lower strength vitamin D preparations, such as drops. The reasoning for the increased use of ampoules is more rapid correction of vitamin D deficiency. Recently, a regulation was implemented by health authorities so that obtaining vitamin D in ampoule form requires prescription from a doctor, which might decrease hypervitaminosis D cases.

The most important finding of the present study is that in Turkey, 25-OHD levels are higher in the first year of life, with significantly lower rates of vitamin D deficiency and insufficiency compared with the other age groups. This favors the vitamin D supplementation programme, launched in 2005, for provision of free vitamin D supplementation to all newborn infants until 1 year of age at a daily dose of 400 U. This programme has been closely supervised by the Ministry of Health, and according to the most recent data, 96.6% of all newborn babies were given vitamin D drops. However, apart from the high success rate of this programme, the prevalence rates of vitamin D deficiency for those younger than 1 year of age still remain above 10% in the Eastern, Southeastern, Black Sea and Mediterranean regions, suggesting that more emphasis be placed on regional differences.

Several recommendations have been made for successful implementation of vitamin D supplementation programmes, which include informing families from birth, providing vitamin D support to all infants, providing vitamin D free of charge, and utilizing the family physician system to monitor usage of vitamin D (15). Turkey is among the European countries with at least 80% compliance with vitamin D supplementation and supervision programme and compared with practices in the UK, the active role of the Ministry of Health is of particular importance for the continuation of the program (15,16). However, despite the high success rate of the programme, some problems still remain. As high as 47% of pediatricians recommend or prescribe vitamin D at the end of the second week, and 19.9% discontinue vitamin D due to reasons such as the presence of a small fontanelle (17). According to Global Consensus recommendations, efforts should be continued to provide 400 international units of vitamin D to all newborns starting from the first day of life, regardless of nutritional status (5,18).

As expected, 25-OHD levels showed inverse correlation with PTH and ALP levels but this was most prominent after 14-19 years of age. ALP levels were not correlated with 25-OHD levels in the pediatric age group, possibly owing to a wide reference range and dynamic temporal changes in ALP concentrations that occur with age during infancy and childhood (19).

### Study Limitations

The data presented in this study were from a private laboratory, which may be a somewhat inappropriate sample to represent a nationwide study. The other limitations were the lack of data regarding the time of day the samples were taken, clinical status, lifestyle and vitamin D intake. The low rate of concurrent PTH and ALP measurement caused difficulties in interpreting the severity of vitamin D deficiency. Despite these limitations, due to the high number of measurements, presence of samples from all regions of the country, and the fact that results show expected differences between genders, seasons and age groups, the authors feel that the present study provides important information on, and relevant insight into, vitamin D status in Turkey.

## Conclusion

In conclusion, with the successful implementation of the vitamin D supplementation programme, Turkey seems to have overcome vitamin D deficiency for those under 1 year of age. However, the positive impact of the program does not continue beyond the first year of life, indicating that vitamin D supplementation may be required in older children and adults. On the other hand, evidence of unnecessary and excessive use of vitamin D supplements is of concern and increased awareness about excess as well as deficient vitamin D levels is also required.

## Figures and Tables

**Table 1:**
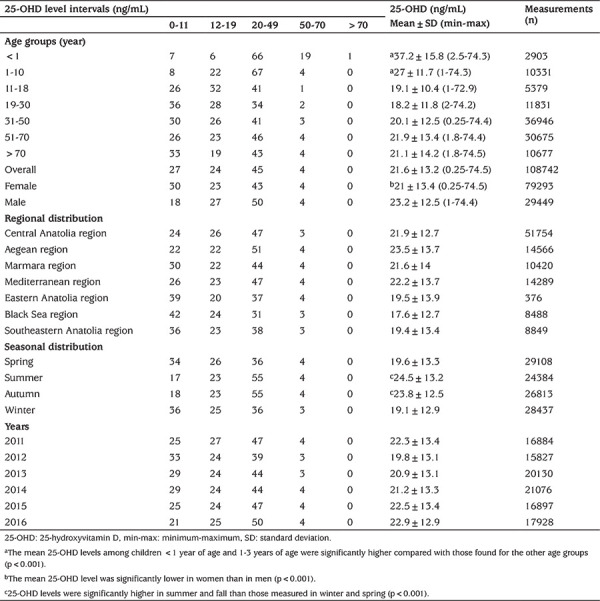
The percentages of serum 25-hydroxyvitamin D levels according to age groups, gender, geographic regions, seasons, and years

**Table 2:**
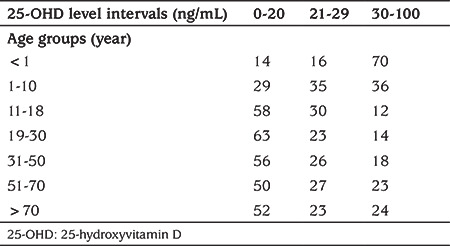
The percentages of vitamin D deficiency, insufficiency and sufficiency based on the Endocrine Society’s recommendations in different age groups

**Table 3:**
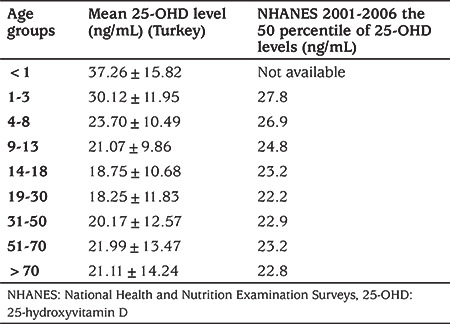
Comparison of the mean serum 25-hydroxyvitamin D levels in our study and the National Health and Nutrition Examination Surveys 2001-2006 according to age groups

**Table 4:**
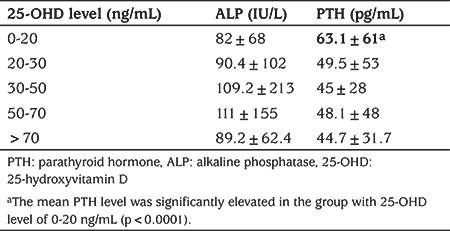
Mean serum levels of alkaline phosphatase and parathyroid hormone according to vitamin D status

## References

[1] Manson JE, Brannon PM, Rosen CJ, Taylor CL (2016). Vitamin D Deficiency - Is There Really a Pandemic?. N Engl J Med.

[2] Basatemur E, Horsfall L, Marston L, Rait G, Sutcliffe A (2017). Trends in the Diagnosis of Vitamin D Deficiency. Pediatrics.

[3] Hatun Ş, Ozkan B, Bereket A (2011). Vitamin D deficiency and prevention: Turkish experience. Acta Paediatr.

[4] Looker AC, Johnson CL, Lacher DA, Pfeiffer CM, Schleicher RL, Sempos CT (2011). Vitamin D status: United States, 2001-2006. NCHS Data Brief.

[5] Munns CF, Shaw N, Kiely M, Specker BL, Thacher TD, Ozono K, Michigami T, Tiosano D, Mughal MZ, Mäkitie O, Ramos-Abad L, Ward L, DiMeglio LA, Atapattu N, Cassinelli H, Braegger C, Pettifor JM, Seth A, Idris HW, Bhatia V, Fu J, Goldberg G, Sävendahl L, Khadgawat R, Pludowski P, Maddock J, Hyppönen E, Oduwole A, Frew E, Aguiar M, Tulchinsky T, Butler G, Högler W (2016). Global Consensus Recommendations on Prevention and Management of Nutritional Rickets. J Clin Endocrinol Metab.

[6] Holick MF, Binkley NC, Bischoff-Ferrari HA, Gordon CM, Hanley DA, Heaney RP, Murad MH, Weaver CM;, Endocrine Society (2011). Evaluation, treatment, and prevention of vitamin D deficiency: an Endocrine Society clinical practice guideline. J Clin Endocrinol Metab.

[7] Ross AC, Manson JE, Abrams SA, Aloia JF, Brannon PM, Clinton SK, Durazo-Arvizu RA, Gallagher JC, Gallo RL, Jones G, Kovacs CS, Mayne ST, Rosen CJ, Shapses SA (2011). The 2011 report on dietary reference intakes for calcium and vitamin D from the Institute of Medicine: what clinicians need to know. J Clin Endocrinol Metab.

[8] Rosen CJ, Abrams SA, Aloia JF, Brannon PM, Clinton SK, Durazo-Arvizu RA, Gallagher JC, Gallo RL, Jones G, Kovacs CS, Manson JE, Mayne ST, Ross AC, Shapses SA, Taylor CL (2012). IOM committee members respond to Endocrine Society vitamin D guideline. J Clin Endocrinol Metab.

[9] The Turkish Ministry of Health Vitamin D Scientific Board Resolutions 2019.

[10] Basatemur E, Hunter R, Horsfall L, Sutcliffe A, Rait G (2017). Costs of vitamin D testing and prescribing among children in primary care. Eur J Pediatr.

[11] Ferrari R, Prosser C (2016). Testing vitamin D levels and choosing wisely. JAMA Intern Med.

[12] Souberbielle JC, Benhamou CL, Cortet B, Rousière M, Roux C, Abitbol V, Annweiler C, Audran M, Bacchetta J, Bataille P, Beauchet O, Bardet R, Benachi A, Berenbaum F, Blain H, Borson-Chazot F, Breuil V, Briot K, Brunet P, Carel JC, Caron P, Chabre O, Chanson P, Chapurlat R, Cochat P, Coutant R, Christin-Maitre S, Cohen-Solal M, Combe C, Cormier C, Courbebaisse M, Debrus G, Delemer B, Deschenes G, Duquenne M, Duval G, Fardellone P, Fouque D, Friedlander G, Gauvain JB, Groussin L, Guggenbuhl P, Houillier P, Hannedouche T, Jacot W, Javier RM, Jean G, Jeandel C, Joly D, Kamenicky P, Knebelmann B, Lafage-Proust MH, LeBouc Y, Legrand E, Levy-Weil F, Linglart A, Machet L, Maheu E, Mallet E, Marcelli C, Marès P, Mariat C, Maruani G, Maugars Y, Montagnon F, Moulin B, Orcel P, Partouche H, Personne V, Pierrot-Deseilligny C, Polak M, Pouteil-Noble C, Prié D, Raynaud-Simon A, Rolland Y, Sadoul JL, Salle B, Sault C, Schott AM, Sermet-Gaudelus I, Soubrier M, Tack I, Thervet E, Tostivint I, Touraine P, Tremollières F, Urena-Torres P, Viard JP, Wemeau JL, Weryha G, Winer N, Young J, Thomas T (2016). French law: what about a reasoned reimbursement of serum vitamin D assays?. Geriatr Psychol Neuropsychiatr Vieil.

[13] Shaw NJ, Mughal MZ (2013). Vitamin D and child health: part 2 (extra-skeletal and other aspects). Arch Dis Child.

[14] Arslan G, Acar S, Nalbantoğlu Ö, et al (2019). Son Beş Yılda Pediatri Polikliniklerine Başvuran Çocuklarda Serum D Vitamini Düzeylerinin Geriye Dönük Olarak Değerlendirilmesi. Journal of Dr. Behçet Uz Children’s Hospital.

[15] Uday S, Kongjonaj A, Aguiar M, Tulchinsky T, Högler W (2017). Variations in infant and childhood vitamin D supplementation programmes across Europe and factors influencing adherence. Endocr Connect.

[16] Uday S, Högler W (2018). Prevention of rickets and osteomalacia in the UK: political action overdue. Arch Dis Child.

[17] Seymen Karabulut G, Hatun Ş, Bideci A, Hasanoğlu E (2016). Attitudes of Pediatricians Regarding Prevention and Treatment of Vitamin D Deficiency. J Clin Res Pediatr Endocrinol.

[18] Yeşiltepe Mutlu G, Hatun Ş (2018). Use of Vitamin D in Children and Adults: Frequently Asked Questions. J Clin Res Pediatr Endocrinol.

[19] Turan S, Topcu B, Gökçe İ, Güran T, Atay Z, Omar A, Akçay T (2011). Serum Alkaline Phosphatase Levels in Healthy Children and Evaluation of Alkaline Phosphatase z-scores in Different Types of Rickets. J Clin Res Pediatr Endocrinol.

